# Effect of Synchronized Laser Shock Peening on Decreasing Defects and Improving Microstructures of Ti-6Al-4V Laser Joint

**DOI:** 10.3390/ma16134570

**Published:** 2023-06-24

**Authors:** Li Zhang, Wentai Ouyang, Di Wu, Liyuan Sheng, Chunhai Guo, Licheng Ma, Zhihao Chen, Zhenkai Zhu, Yongxiao Du, Peijuan Cui, Zhanlin Hou, Wenwu Zhang

**Affiliations:** 1 School of Materials Science and Chemical Engineering, Ningbo University, Ningbo 315211, China; zhangli21@nimte.ac.cn (L.Z.);; 2 Key Laboratory of Aero Engine Extreme Manufacturing Technology of Zhejiang Province, Ningbo Institute of Materials Technology and Engineering, CAS, Ningbo 315201, China; ouyangwentai@nimte.ac.cn (W.O.);; 3 Shenzhen Institute, Peking University, Shenzhen 518057, China; 4 PKU-HKUST Shenzhen-Hongkong Institution, Shenzhen 518057, China; 5 Beijing Institute of Precision Mechatronics and Controls, Beijing 100076, China

**Keywords:** laser shock peening, welding defect, microstructure, mechanical properties, Ti-6Al-4V laser joint

## Abstract

Repairing processing is a significant method for damaged high-cost Ti-6Al-4V components to decrease economic loss, which usually utilizes a welding technique. For a large-size structural component, welding processing is commonly completed in air conditioning, which makes it difficult to avoid welding defects. To this end, an appropriate matching technique is important for improving welding performance. In the present research, asynchronized laser shock peening (ALSP) and synchronized laser shock peening (SLSP) techniques were utilized to decrease the influence of macro welding defects on laser-welded Ti-6Al-4V joints. The results show that SLSP has a greater effect on inducing surface plastic deformation on Ti-6Al-4V joints with a pitting depth of more than 25 microns while ALSP can lead to a pitting depth of about 15 microns. Through micro-CT observation a long hot crack exists in the central area of as-welded joints with a length of about 2.24 mm, accompanied by lots of pores in different sizes on double sides. After ALSP processing, some pores are eliminated while others are enlarged, and one-side crack tips present closure morphology. However, some microcracks exist on the side-wall of hot cracks. With the influence of SLSP, significant shrinkage of pores can be observed and both sides of crack tips tend to be closed, which presents a better effect than ALSP processing. Moreover, greater effects of grain refinement and thermal stress release could be achieved by SLSP processing than ALSP, which can be ascribed to dynamic recrystallization. For the as-welded joint, the ultimate tensile strength (UTS) and elongation (EL) values are 418 MPa and 0.73%, respectively. The values of UTS and EL in the ALSP processed joint are increased to 437 MPa and 1.07%, which are 4.55% and 46.48% higher than the as-welded joint, respectively. Such values after SLSP processing are 498 MPa and 1.23%, which are 19.14% and 68.49% higher than the as-welded joint, respectively.

## 1. Introduction

As a kind of famous alloy, titanium alloys have many outstanding features, such as highly specific strength, good resistance and excellent mechanical properties, and have been widely used in marine and aerospace industries [1,2]. Among titanium alloys, Ti-6Al-4V has the most widespread application due to its balanced performance, which has been usually used in advanced components [3,4]. Owing to the high cost of many Ti-6Al-4V manufactured components, their failure can bring enormous economic losses. Therefore, it is significant for repairing the damaged area in these failure components to increase their service life [5,6].

Welding is a commonly used repairing method in many fields, which repairs failure components by connecting or filling the damaged area [7,8,9]. Many welding techniques have been developed currently, which includes explosive welding, friction welding, TIG welding and laser welding, etc. [10,11,12,13]. Among them, it is difficult for explosive and friction welding techniques to achieve repairing, especially in components with complex structures [13,14]. TIG welding is a useful method for repairing components with various shapes, but its large fusion and heat-affected zones are detrimental to welding the material with high chemical activity [15]. Compared to the previously mentioned welding techniques, laser welding is revolutionary welding that can adjust the width and depth of a molten pool by adjusting the power and diameter of a laser beam, which could meet different demands of repairing with the combination of automation [16,17,18].

However, Ti-6Al-4V is a kind of hard-to-weld material because of its specific properties, including high melting temperature, low thermal conductivity and strong chemical activity. Feng et al. [19] reported the incidental formation and propagation of cracks during the welding process without effective protection. Tsai et al. [20] reported the highly reactive property of Ti-6Al-4V alloy with atmospheric gases in a molten pool, which could cause severe embrittlement. Moreover, due to the rapid solidification rate and high temperature gradient, hot cracking and pore defects usually occur in the laser welding process [21,22,23]. The combined characteristics of Ti-6Al-4V alloy and laser welding process dramatically increase the risk of welding defects in practical applications, especially for the large-size structural parts which need to be repaired in air conditioning. To this end, it is important to achieve shrinkage of welding defects.

Many researchers have applied external fields to the laser welding process to influence the behavior of the molten pool. Hu et al. [22] and Fritzsche et al. [24] reported the effect of an electromagnetic field on decreasing the porosity formed during the laser welding process. However, this method needs to apply an electric current on the component, which would influence the matrix and even lead to damage. Chen et al. [18,25,26] and Bachmann et al. [27] applied magnetic fields to the laser welding process and also obtained the effect of decreasing defects, but the applying method and effective influencing area of the magnetic field limit its application. For large-size structural parts, such as the warship shell, it is difficult to apply such an external field during the welding process. Besides these external fields applied during the welding process, surface micro-deformation is a considerable method to reduce welding defects after the welding process. Wang et al. [28] reported the influence of shot peening on decreasing defects of Al5052/Ti-6Al-4V joints due to the deformation effect. As reported by Damon et al. [29], shot peening could decrease 15–30% porosity of laser-melting material. Wu et al. [30] also presented the effect of shot peening on hindering crack nucleation and propagation nearing pores. However, it is also inappropriate for applying shot peening in large parts due to the problem of collecting pellets and avoiding pollution.

As an advanced surface strengthening technique, laser shock peening (LSP) has attracted widespread attention due to its high energy density, ultra-high strain rate and processing controllability. Since the strengthening effect is achieved by laser beam, it can be flexibly applied to various situations with the combination of automation, such as complex structure, precise components and large-size structural parts. DU Plessis et al. [31] reported the pore closure effect of LSP, and Maleki et al. [32] reported a 62% reduction of porosity induced by LSP, which is much higher than reported in the research on shot peening. Zhou et al. [33] numerically studied the mechanism of compressive pressure applied on pores, and the results show that the plastic deformation driven by shock waves contributes to the repairing of defects, which is closely connected to the maximum pressure intensity. Such result is also reported by Tong et al. [34]. Therefore, LSP is a wise method for reducing welding defects and improving the performance of Ti-6Al-4V laser joints with welding defects. However, previous research on LSP disregarded the relatively extreme situation of the simultaneous existence of macrocracks and pores in welding joints, which could possibly exist in the joint of large-size structural parts due to the higher solidification rate and higher temperature gradient. The much larger size of the defect determines the higher energy needed to achieve defect decreasing. Although LSP processing could be asynchronously applied on both sides of the material to increase its effect, a more preferable method needs to be developed to further increase the macro-defect decreasing effect. Some researchers have proposed a two-sided laser shock peening processing with a synchronous laser beam in the same position, which could apply a much higher residual compressive stress on materials compared with the asynchronous LSP processing using the same energy [35,36]. However, its actual effect on decreasing the macro defect of Ti-6Al-4V alloys has not been experimentally studied.

In this study, a synchronized laser shock peening (SLSP) processing was developed to treat Ti-6Al-4V laser joints, accompanied by macrocracks and pores, on both sides simultaneously. A comparative study of SLSP and traditional asynchronized laser shock peening (ALSP) processing was performed to explore their effects on decreasing welding defects and improving microstructures and mechanical properties were investigated to present their practical effect.

## 2. Experimental Procedure

### 2.1. Sample Preparation

**Figure 1:**
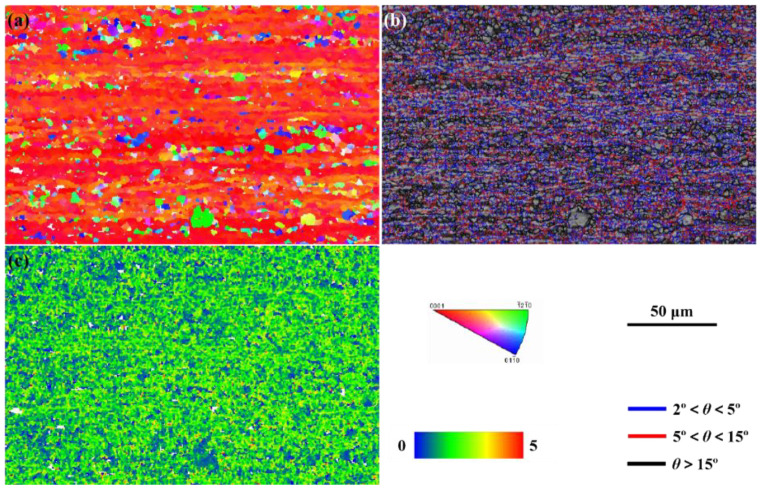
Microstructure of the as-received Ti-6Al-4V alloy: (**a**) Inverse pole figure map; (**b**) Grain boundaries; (**c**) Distribution of the kernel average misorientation.

The Ti-6Al-4V alloy rolled plates were used in this study. The as-received Ti-6Al-4V alloy plates had the size of 150 mm × 150 mm × 3 mm, which were cut into the samples of 50 mm × 40 mm × 3 mm by wire-cut electrical discharge (WED) for use in this research. The surfaces and side-walls were polished with sandpaper and cleaned with ethanol for 15 min to ensure surface quality, avoiding additional disturbance introduced in specimen preparation. The microstructure of the as-received Ti-6Al-4V alloy is shown in Figure 1, which is the typical rolled microstructure. Figure 1a reveals a typical rolled TC4 microstructure with obvious texture characteristics, and most of the crystals are inclined to grow along {0001} a crystal plane family. Moreover, the grains also prefer to grow along the rolling direction. Figure 1b,c indicate the high-density low-angle grain boundaries and misorientation in material, indicating a large density of dislocations.

### 2.2. Laser Welding Process and Laser Shock Peening Process

**Figure 2:**
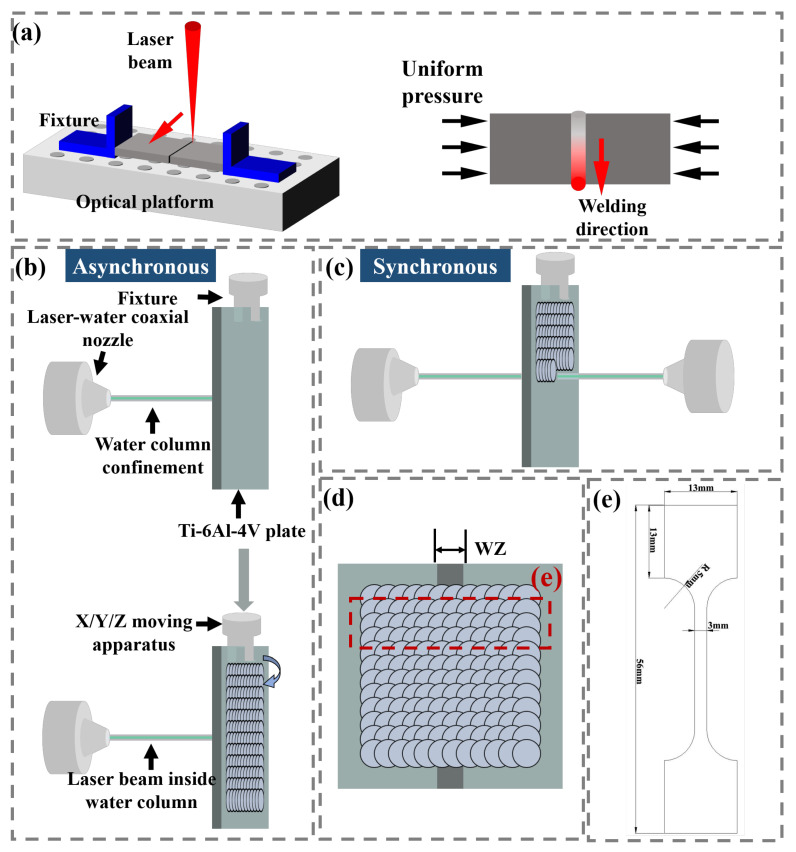
Schematic diagrams of (**a**) LW, (**b**) ALSP, (**c**) SLSP, (**d**) LSP processing path and tensile test sampling method; (**e**) Tensile test specimen with specific dimensions.

A continuous wave (CW) fiber laser (IPG Photonics, Newton, MA, America) was used to weld butted Ti-6Al-4V plates with a linear path. The laser welding (LW) processing was completed on an optical platform, and a pair of fixtures were used to provide uniform pressure for the purpose of making two plates butted closely, as shown in Figure 2a. Both sides of the thin plate were consistently subjected to uniform pressure during welding processing, and the former welding area has solidified. The CW fiber laser system used in LW processing includes a six-axis KUKA robot (KUKA company, Augsburg, Germany), a 4000 W fiber laser set with 1070 nm wavelength and a laser welding head. The processing parameters were set as laser power of 1200 W, scanning speed of 10 mm/s and spot diameter of 1 mm, respectively.

In the present research, laser shock peening experiments were performed using a Nd:YAG laser (Shanghai Institute of Optics and Fine Mechanics, Chinese Academy Sciences, Shanghai, China) at 10 Hz and 1064 nm wavelength. A self-developed dual-beam synchronous LSP system is used to illuminate the laser beam on the opposite sides of the plate synchronously and move the plate according to the program. The detailed laser processing parameters are as follows: laser density of 5.26 GW/cm^2^, pulse duration is 8 ns and the laser beam diameter of 2 mm and overlap ratio (along both X and Y directions) of 50%. The self-developed laser–water coaxial supply system is used to realize the constraint layer, and the Ti-6Al-4V plate is clamped with a fixture on the X/Y/Z mobile device. As shown in Figure 2b, the LSP process on both sides of the plate is completed asynchronously; this process method is defined as an asynchronous laser shock peening (ALSP) process. Correspondingly, the LSP process completed simultaneously on both sides is defined as the simultaneous laser shock peening (SLSP) process, and the schematic diagram is shown in Figure 2c. Figure 2d shows the spot overlap method, and the WZ, HAZ and part of the matrix were contained in the LSP region.

### 2.3. Microstructural Characterization

The specimens used for cross-section microstructure analysis were cut from a laser joint with LSP and without LSP, respectively. The samples were embedded in thermosetting metallographic resin and then ground with silicon carbide (SiC) sandpapers from 280 to 2000. Then the sample was polished with a diamond polishing paste with a particle size of 1 μm, and the polished samples were etched with Keller reagent to show the boundary. A VX-X200K laser confocal scanning microscope (LCSM) (KEYENCE Corporation, Shanghai, China) was used to obtain an optical microscopic (OM) image and surface profile, and the microstructures characterized on the cross-section of laser joints and corresponding fracture surface were obtained by FEI Quanta FEG 250 scanning electron microscope (SEM) (FEI company, Hillsboro, OR, America). Electron backscatter diffraction (EBSD) analysis was assembled on Verios G4 UC scanning electron microscope (SEM) (Thermo Fisher Scientific, Waltham, MA, America), and its inverse pole figure (IPF), grain boundary and recrystallization structure were characterized to explore the effect of LSP processing on microstructure evolution. The EBSD observation samples were prepared by using an EP-06X electrolytic polishing apparatus (Yongying Company, Ningbo, China), and the polishing etchant we used was a mixture, containing 10 mL hydrofluoric acid, 60 mL hydrogen peroxide and 30 mL deionized water. In order to precisely present the effect of LSP on decreasing welding defects in a larger area, an Xradia-610 micro-CT (ZEISS Comopany, Oberkochen, Germany) was used to construct pores and cracks inside joints with the dimensions of 3 mm × 5 mm × 8 mm.

### 2.4. Mechanical Tests and Characterization

The tensile specimen sampling method and schematic diagram are shown in Figure 2d and Figure 2e, respectively. The tensile test piece was prepared by a CNC ultra-high pressure water cutting machine (NingBo Zhongyuan Machine Co., Ltd., Ningbo, China). The side-wall of the test piece was relatively smooth without grooves, and the tensile test could be completed directly. The tensile test was performed on a Zwick/Roell Z030 (Zwick Roell, Ulm, Germany) universal material testing machine at a tensile speed of 0.5 mm/min. Before the tensile test, the width and thickness of the tensile specimen were accurately tested to obtain accurate tensile stress. Three tensile samples were taken for each state to ensure the stability of the testing results.

## 3. Results and Discussion

### 3.1. Surface Topography Evolution Induced by LSP Processing

**Figure 3:**
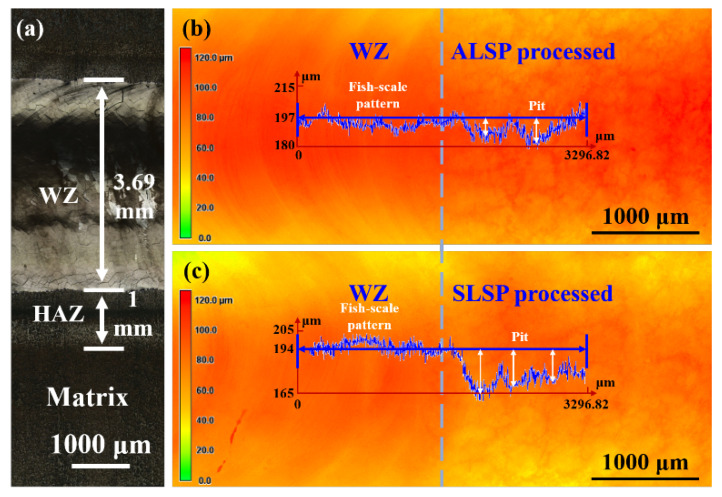
Surface analysis of laser joints with different states: (**a**) Macroscopic OM image; Macroscopic 3D topography of the junction area containing the as-welded laser joint and (**b**) ALSP-processed joint or (**c**) SLSP-processed joint (Dashed line separating the WZ and LSP processed area).

Since the effect of LSP processing is closely related to plastic deformation capacity, the surface morphologies of laser-welded joints processed by ALSP and SLSP are characterized, and the results are presented in Figure 3. As shown in Figure 3, it is obvious that the as-welded joint presents the typical welding feature of fish-scale patterns. The welding zone (WZ) and heat-affected zone (HAZ) of an as-welded joint are about 3.69 mm and 1 mm, respectively. The width of HAZ is identified by surface color variation induced by heat input. The detailed surface topographies on the junction area of ALSP- and SLSP- processed WZ are shown in Figure 3b,c, and the scale in height has been normalized into the same. The curves in Figure 3b,c are related to their specific surface topography, respectively, which represent the variation of depth along the blue arrow. Obvious elimination of the fish-scale pattern could be observed from the LSP-processed WZ surface and pitting morphology present on the surface, which should be ascribed to the plastic deformation induced by LSP processing. Consequently, the height of WZ has been slightly decreased by such plastic deformation, which could be found from the variation of the height of the curve. Moreover, a deeper pit can be achieved by SLSP processing, by comparing the surface topographies of ALSP- and SLSP-processed WZ. The height difference between the top and bottom of the pit induced by ALSP processing is about 15 microns, while that of SLSP is more than 25 microns. This indicates the greater effect of SLSP processing on inducing plastic deformation, which might have a better effect on promoting microstructure evolution and defect decreasing.

### 3.2. Welding Defect Evolution Induced by LSP Processing

**Figure 4:**
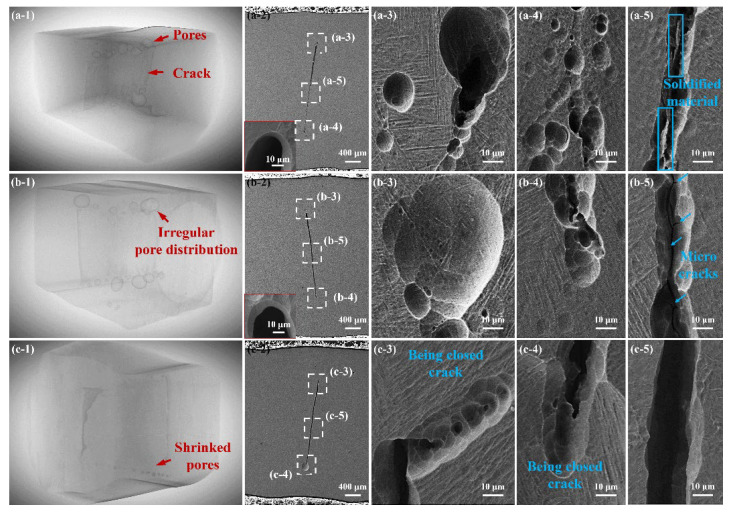
Hybrid analyses on welding defects of the joints with different states: (**a-1**–**a-5**) As-welded joint; (**b-1**–**b-5**) ALSP-processed joint; (**c-1**–**c-5**) SLSP-processed joint; (**a-1**,**b-1**,**c-1**) Macroscopic CT view of part of the joint containing WZ, HAZ and the matrix; (**a-2**,**b-2**,**c-2**) Macroscopic SEM image of WZ (inset image in (**a-2**) shows the typical crack edge of an as-welded joint, and that in (**b-2**) shows the edge on the top side of the crack of an ALSP-processed joint); (**a-3**,**b-3**,**c-3**) Microscopic SEM image of defects on the top side of the crack; (**a-4**,**b-4**,**c-4**) Microscopic SEM image of defects on the bottom side of the crack; (**a-5**,**b-5**,**c-5**) Microscopic SEM image showing the middle area of macro crack.

**Figure 5:**
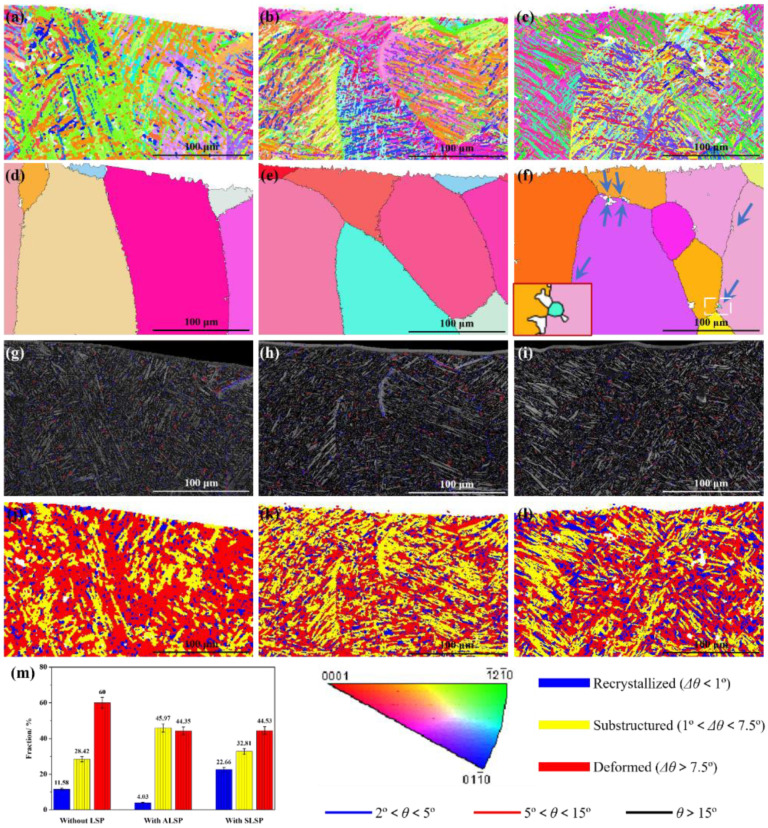
EBSD analyses on the top region of the joints with different states: IPF maps of (**a**) as-welded joint; (**b**) ALSP-processed joint; (**c**) SLSP-processed joint; Prior β grain reconstruction maps of (**d**) as-welded joint; (**e**) ALSP-processed joint; (**f**) SLSP-processed joint; Grain boundary distribution on band contrast maps of (**g**) as-welded joint; (**h**) ALSP-processed joint; (**i**) SLSP-processed joint; Recrystallized maps of (**j**) as-welded joint; (**k**) ALSP-processed joint; (**l**) SLSP-processed joint; (**m**) Fraction of recrystallized structure.

Due to the great influence of welding defects on mechanical performance, the effect of ALSP and SLSP processing on macro defects is shown through two modes of cross-sectional view and overall section construction, as shown in Figure 4. Figure 4(a-1) demonstrates the construction of a part of an as-welded joint, where it is obvious that a crack exists in the central area of the joint and many pores with diverse sizes distribute adjacent to the double sides of the crack. Moreover, the length of the crack keeps relatively constant in the as-welded joint, which should be ascribed to the hot crack induced by the tensile deformation during the solidification process. The sizes of pores and the crack could be revealed by comparing with the thickness of the sample of 3 mm. The edge of the crack presents an approximately circular morphology, as shown in the inset image of Figure 4(a-2). The cross-sectional view also reveals the pores on both of the top and bottom side of the crack, and the length of the crack is about 2.24 mm, as shown in Figure 4(a-2)–(a-4). The size of the pores on the top side is larger than 50 microns while that on the bottom side is about 10 microns, as shown in Figure 4(a-3). Although the sizes of the pores on the bottom side are smaller, their numbers are much larger than the top side, as shown in Figure 4(a-4). As shown in Figure 4(a-5), some connections still exist inside the crack, which seem to be the solidified material left after crack generation. Additionally, the laths on different sides of the crack show greatly diverse morphology, which belongs to different prior β grains.

After ALSP processing, the distribution of pores becomes irregular, as shown in Figure 4(b-1). Pores are eliminated in some areas through the view obtained by CT, however, some pores are enlarged. Moreover, most of the pores are close to the crack. From SEM images, it could be found that the pore on the top side of the crack is larger than that of the as-welded joint, but the pores on the bottom side are almost disappeared, leaving the macrocrack with a microcrack inside, as shown in Figure 4(b-3,b-4). Though the top edge of the crack still demonstrates the circular feature, the bottom edge is no longer round, revealing closure morphology at the bottom edge. Furthermore, the connection inside the crack disappeared in the ALSP-processed joint, which could be broken down by ALSP processing, and some microcracks exist on the side-wall of the crack, as shown in Figure 4(b-5). Such microcracks should be attributed to the deformation induced by ALSP processing and are detrimental for undertaking loads applied on the joint. Furthermore, the irregular decreasing of defects indicates the uneven deformation behavior induced by ALSP [34].

For the SLSP-processed joint, significant shrinkage of pores could be observed from the view of CT, and no large pores exist on either side of the crack, as shown in Figure 4(c-1). Especially on the top side, pores are almost eliminated by the influence of SLSP processing. Different from the as-welded and ALSP-processed joints, the pores in the SLSP- processed joint are away from the crack, which seems to shrink toward the central area. Moreover, the top edge of the crack is deviated from the previous site, which turns to the side presently, and its interior demonstrates a trend of closure, as shown in Figure 4(c-3). Similarly, the bottom edge also presents a closure tendency, since part of the edge is connected in deeper depth, as shown in Figure 4(c-4). Both of the edges on the top and bottom sides are no longer circular, and pores have disappeared from the surrounding areas. It could be deduced that such results are induced by plastic deformation induced by SLSP processing. Different from the as-welded joint and ALSP-processed joint, the central area of the crack seems to be enlarged, and no connections and microcracks could be observed from the crack interior, as shown in Figure 4(c-5). Such variation could be ascribed to the simultaneous compression from both sides of the plate to the interior of the macrocrack. By comparing the results of welding defects, it can be indicated that ALSP and SLSP processing have different deformation modes on a welding joint, and thus could achieve different effects on decreasing the influence of welding defects.

### 3.3. Microstructure Evolution Induced by LSP Processing

Based on the welding defect situation, the effective part which can undertake a load is the area between the surface and crack. Therefore, the strengthening of such an area is important for improving the mechanical performance of laser joints with macro welding defects. Moreover, as a surface-strengthening technique, the most effective strengthening area of LSP is the area near the surface, which is consistent with the area that needs to be strengthened. Figure 5 reveals the EBSD analysis results in the top region of joints with different states. As shown in Figure 5a, the laths in the top region of the as-welded joint present typical basket-weave characteristics, and several areas with similar lath morphologies could be observed, accompanied by some small laths which interleave in long-length laths. According to the reconstruction result calculated by MATLAB software, these areas should be prior β grains with relatively large sizes, as shown in Figure 5d. Although in the same prior β grain, the crystallographic orientation of laths is greatly diverse. Through the grain boundary distribution of the as-welded joint shown in Figure 5g, high- angle grain boundary (HAGB) is the main interface separating the laths in the same prior β grain, and some low-angle grain boundaries (LAGB) uniformly distribute in the top area. Figure 5j presents the distribution of recrystallized, substructured and deformed laths in the top region; the related fraction is shown in Figure 5m. It can be seen that deformed structures occupy the principal position, which should be ascribed to the high-level thermal stress induced by the rapid solidification of laser welding processing [37,38]. Moreover, 11.58% of recrystallized structures and 28.42% of substructured structures could be found from the top area, respectively. The uneven distribution of these structures indicates the uneven influence of thermal stress [39].

After exerting ALSP on the joint, prior β grains are slightly refined, as shown in Figure 5e. However, as shown in Figure 5b, the growth directions and crystallographic orientation of laths in the same prior β grain tend to be similar, compared with the as-welded joint. Such morphology may lead to crystal anisotropy within the prior β grain scale. Based on the grain boundary distribution map, more coarser laths in the adjacent area between prior β grains could be observed with the influence of ALSP processing, as shown in Figure 5h. Moreover, in the top region of the ALSP-processed joint, the fractions of recrystallized, substructured and deformed structures are 4.03%, 45.97% and 44.35%, respectively. It is obvious that the fractions of deformed and recrystallized structures are decreased while that of substructured structures is increased with the effect of ALSP processing. Such a result indicates that ALSP processing can decrease the thermal stress so that the fraction of deformed structure is reduced. However, lots of dislocations could be introduced into the laths without a severe influence of thermal stress, such as recrystallized structure. As a result, the fraction of recrystallized structure is also reduced.

With the exertion of SLSP, a great promotion of microstructure evolution could be observed from Figure 5c,f,i,l. Firstly, the laths in the top region are obviously refined, especially in thickness, compared with as-welded and ALSP-processed joints, as shown in Figure 5c. Moreover, prior β grains are also refined with the influence of SLSP processing, and some dynamic recrystallized (DRX) grains could be widely found from prior β grain boundaries, as indicated by the arrow (inset image is an enlarged image of DRX grains). Such morphology seldom exists in as-welded and ALSP-processed joints, which indicates the greatest effect of SLSP on promoting DRX. Furthermore, the band contrast (BC) value [40,41] in lots of laths is lower (lighter color), indicating the greater DRX effect, as shown in Figure 5i. Moreover, less LAGBs distribute in the top region of the SLSP-processed joint, which could be absorbed during the DRX process. As shown in Figure 5l,m, a significant increase in recrystallized structure and a decrease in deformed structure can be simultaneously observed, and the fraction of substructured structure keeps relatively constant. The fractions of recrystallized, substructured and deformed structures are 22.66%, 32.81% and 44.53%, respectively. Compared with the ALSP-processed joint, the fraction of recrystallized structure is dramatically higher while the fraction of substructured structure is lower. However, the fractions of deformed structure with the two LSP processing modes are similar. Such a phenomenon indicates the better effect of SLSP on decreasing the thermal stress induced by laser welding processing through the DRX process.

**Figure 6:**
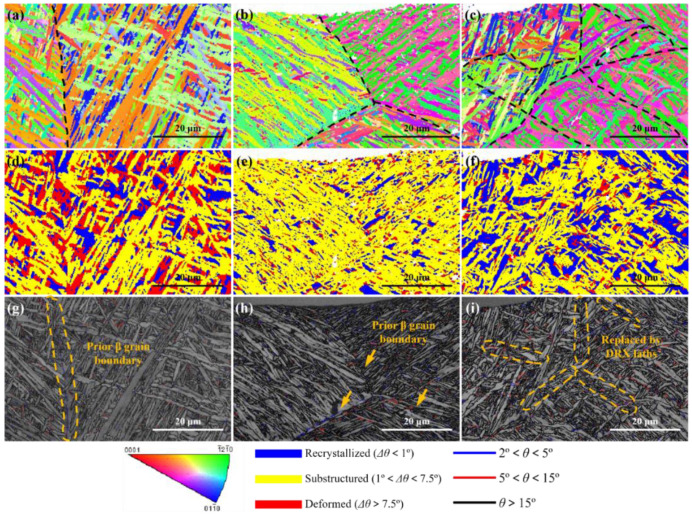
Detailed EBSD analyses on the top layers of joints with high magnification: IPF maps of (**a**) as-welded joint; (**b**) ALSP-processed joint; (**c**) SLSP-processed joint; Recrystallized maps of (**d**) as-welded joint; (**e**) ALSP-processed joint; (**f**) SLSP-processed joint; Grain boundary distribution on band contrast maps of (**g**) as-welded joint; (**h**) ALSP-processed joint; (**i**) SLSP-processed joint.

In order to further explore the effects of different LSP processing modes on microstructure evolution of Ti-6Al-4V laser joints, detailed EBSD analyses were performed on the top layers of joints with different states. As shown in Figure 6a, a distinct prior β grain boundary of an as-welded joint could be observed, and the grain boundary distribution map reveals that LAGB acts as a grain boundary, as shown in Figure 6g. Some deformed structures with intensive crystal defects distribute in the top layer, as shown in Figure 6d. The difference in deformed fraction between Figure 5j and Figure 6d can be attributed to the scale effect of characterization and calculation of EBSD processing software.

Figure 6b displays the IPF map on the top layer of the ALSP-processed joint, where it is apparent that the basket-weave feature is weakened due to the plastic deformation induced by ALSP processing, which forms a lamellar structure and could be detrimental for the ductility of the joint. The growth directions of laths in the same prior β grain tend to be similar, and the crystal orientations of these laths are greatly different from the as-welded joint and also tend to be consistent, which indicates the tilting effect of ALSP processing on crystals. Moreover, significant refinement of lath was achieved by ALSP processing, accompanied by the normalization transformation of recrystallized and deformed structures towards substructured structure, as shown in Figure 6e,h. However, the refinement of lath structure is uneven, and multiple relatively coarse laths still aggregate in the prior β grain. The decrease of deformed structure and refinement of lath indicate the occurrence of DRX, but a low fraction of recrystallized structure demonstrates the incomplete procedure. Moreover, the prior β grain boundary is still obvious in the ALSP-processed joint, which is similar to an as-welded joint, but the boundaries are usually composed of LAGB, as shown in Figure 6h.

For the SLSP-processed joint, typical basket-weave characteristics could be seen from the top layer, and the shape of laths presents twisting morphology, as shown in Figure 6c. This result demonstrates the preferable effect of SLSP processing on promoting deformation of laths without transformation to a lamellar structure, which should be ascribed to its specific deformation mechanism. With the influence of this deformation, a considerable proportion of laths have been transformed into recrystallized laths (Figure 6f), containing prior β grain boundaries. A large proportion of prior β grain boundaries are replaced by DRX laths with ultra-fine size, which might have greater capability for coordinate deformation. Such a transformation from grain boundary to DRX laths can also decrease crystal defects induced by laser welding.

### 3.4. Mechanical Properties and Fracture Analyses

**Figure 7:**
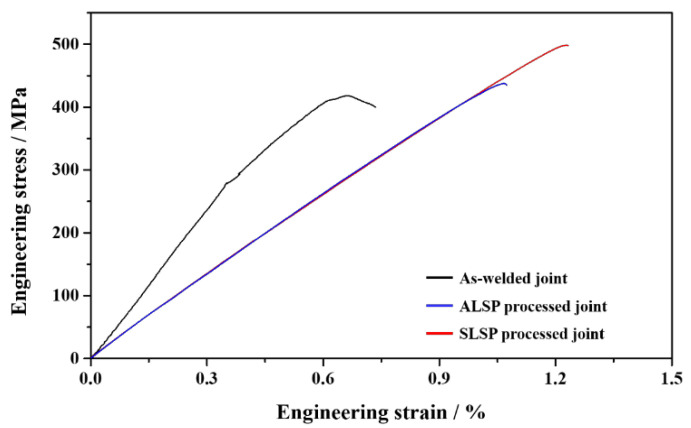
Engineering stress–strain curves of the as-welded joint, ALSP- and SLSP-processed joint.

The typical engineering stress–strain curves of joints with different states are displayed in Figure 7. The ultimate tensile strength (UTS) of the as-welded joint shown in Figure 7 is 418 MPa and its total elongation (TEL) is 0.73%. These are much lower than standard mechanical properties due to the existence of macro welding defects. With the influence of ALSP processing, the values of UTS and TEL reach 437 MPa and 1.07%, respectively. Compared to an as-welded joint, the results of UTS and TEL have increased by 4.55% and 46.58%, respectively. It can be seen that the ductility is dramatically increased although the increase in strength is a little. Such results should be ascribed to the combined results of inadequate closure of pores and crack tip, slight grain refinement and released thermal stress. After SLSP processing, great simultaneous increase of strength and ductility could be achieved, and the UTS and TEL are 498 MPa and 1.23%, which are 19.14% and 68.49% higher than an as-welded joint, respectively. This great improvement could be attributed to the greater defect closure effect, grain refinement and effective thermal stress release. The UTS and TEL values of joints averaged from three samples of each parameter are displayed in Table 1. It should be noted that the UTS and TEL are still much lower than the welding or melting parts with high-performance structure [42,43].

**Figure 8:**
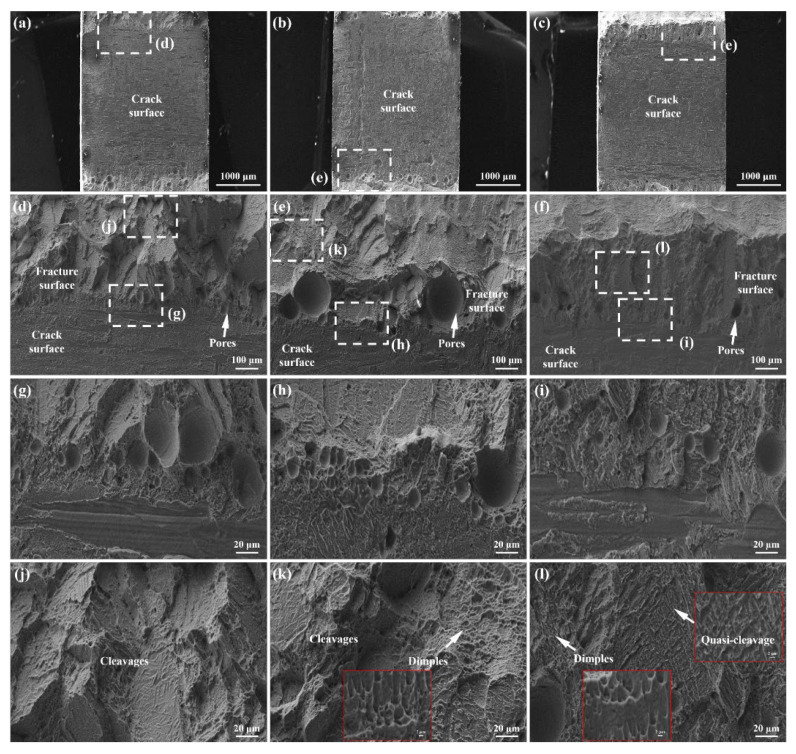
SEM observations on the tensile fracture morphologies of the joints with different LSP processes: (**a**) Macroscopic image of the fracture surface of as-welded joint; (**b**) Macroscopic image of the fracture surface of ALSP-processed joint; (**c**) Macroscopic image of the fracture surface of SLSP-processed joint; (**d**) Fracture morphology near the crack of as-welded joint; (**e**) Fracture morphology near the crack of ALSP-processed joint; (**f**) Fracture morphology near the crack of SLSP- processed joint; (**g**,**j**) Enlarged fracture morphology of as-welded joint; (**h**,**k**) Enlarged fracture morphology of ALSP-processed joint; (**i**,**l**) Enlarged fracture morphology of SLSP-processed joint.

Further observations on fracture morphologies of joints are displayed in Figure 8. Through macro images, it could be found that the actual area undertaking load distributes on both sides of the fracture, accompanied by a coarse surface, as shown in Figure 8a–c. The central area of the fracture presents relatively smooth characteristics, which is the surface of the macrocrack formed in laser welding. Figure 8d shows the junction area of the as-welded joint containing part of the crack surface and fracture surface. A large number of pores exist in this area, and the enlarged view reveals the pore size ranges from 3.34 μm to 35 μm, as shown in Figure 8g. Moreover, the enlarged image of the fracture surface presents obvious cleavage morphology, as shown in Figure 8j. As a result, the fracture mode in the as-welded joint can be classified as a cleavage fracture, indicating the brittle fracture mode of the as-welded joint. For the ALSP-processed joint, Figure 8e displays the junction area containing a part of the crack surface and fracture surface. It is obvious that several large pores exist in the junction area with a size of about 183.35 μm, accompanied by some pores in smaller sizes, as shown in Figure 8h. Compared with the as-welded joint, the number of pores in the ALSP-processed joint is obviously less. Furthermore, typical cleavage morphology can be also found on the fracture surface of the ALSP-processed joint. Meanwhile, the dimples abound in this area, which indicates better ductility with the influence of ALSP processing, as shown in Figure 8k and the inset image. With the influence of SLSP processing, it is obvious that the number and size of pores are dramatically decreased in the junction area, compared with the as-welded joint and ALSP-processed joint, as shown in Figure 8f,i. In addition, no obvious cleavage step could be found on the fracture surface. As shown in Figure 8l and the inset images, a large number of dimples could be found in the fracture surface of the SLSP-processed joint. Meanwhile, quasi-cleavage characteristics could be observed from the fracture surface, revealing the best ductility.

### 3.5. Influence of LSP Processing on Decreasing Welding Defects

Due to the existence of macro defects in laser joints, the effective area undertaking the responsibility of structural material is dramatically decreased. Moreover, the presence of pores and cracks can greatly increase the sensitivity of the crack, which promotes its initiation and propagation [30]. Therefore, decreasing defects plays a vital role in improving the performance of laser joints. According to previous research [28], surface plastic deformation can lead to the decrease of defects. Since pores and cracks have an unstable structure, it is straightforward for deformation to change the surrounding structure of these defects. Consequently, the material on the upper side can be squeezed towards the interior of the pore, and the pore with a large size could be segmented into many small pores while small-size pores would be eliminated, therefore decrease the porosity [44]. The obvious pore shrinkage and crack closure induced by SLSP processing could be ascribed to such an effect, as shown in Figure 4(c-1–c-5). The closing mechanism on both sides of the crack is similar to the mechanism of decreasing porosity. The different effects of ALSP and SLSP processing on defects should be attributed to the weaker capability of ALSP on plastic deformation due to its deformation mechanism. Moreover, LSP processing has a significant effect on applying residual compressive stress within a considerable depth of material, which is beneficial for inhibiting the propagation of cracks [30,32,45]. The hybrid effects can help to increase the mechanical performance of the joint with macro defects.

However, the present research neglects the evolution of microstructure and residual stress near defects, which is significant for thoroughly exploring the mechanism of defect restraint. These results would be supplemented in future to sufficiently reveal the effect of LSP processing with different modes on welding defects.

## 4. Conclusions

In the present research, laser welding processing was used to repair the damaged Ti-6Al-4V plates through double sides, and ALSP and SLSP processing were utilized to decrease welding defects and increase microstructures of Ti-6Al-4V laser joints. The influence of ALSP and SLSP on plastic deformation, welding defects, microstructure evolution and mechanical property was researched to explore the preferable method on improving the performance of Ti-6Al-4V laser joints with macro welding defects. Some conclusions could be summarized as follows:

(1) Surface topographies of ALSP and SLSP processing show that SLSP can cause surface plastic deformation on Ti-6Al-4V joints with a pitting depth of more than 25 microns while that of ALSP is about 15 microns.
(2) A long hot crack can be found from the central area of an as-welded joint through micro-CT with a length of about 2.24 mm, accompanied by many pores of different sizes on double sides. With the influence of ALSP processing, part of the pores is eliminated while others are enlarged, and the one-side crack tip presents closure morphology. However, some microcracks exist on the side-wall of the hot crack. After SLSP processing, great shrinkage of pores can be achieved and more obvious closure tendency can be observed from both sides of crack tips.
(3) Greater effects of grain refinement and thermal stress release could be achieved by SLSP processing than ALSP processing, which can be ascribed to dynamic recrystallization.
(4) For the as-welded joint, the UTS and EL values are 418 MPa and 0.73%, respectively. The values of UTS and EL in the ALSP-processed joint are increased to 437 MPa and 1.07%, which are 4.55% and 46.48% higher than the as-welded joint, respectively. The values of UTS and EL after SLSP processing are 498 MPa and 1.23%, which are 19.14% and 68.49% higher than the as-welded joint, respectively.

## Figures and Tables

**Table 1:** Detailed tensile properties of joints with different LSP processing.

Sample	UTS (MPa)	TEL (%)
As-welded joint	426 ± 8	0.87 ± 0.15
ALSP-processed joint	437 ± 2	1.12 ± 0.05
SLSP-processed joint	479 ± 19	1.19 ± 0.04

## Data Availability

Data are contained within the article.
